# Perception and Attitudes of Dental Professionals on Teledentistry: A Cross-Sectional Study

**DOI:** 10.1055/s-0044-1801301

**Published:** 2025-03-12

**Authors:** Yousuf Moosa, Lakshman Perera Samaranayake, Pagaporn Pantuwadee Pisarnturakit

**Affiliations:** 1 Department of Community Dentistry, Faculty of Dentistry, Chulalongkorn University, Bangkok, Thailand; 2 Department of Dentistry, University of Hong Kong, Hong Kong; 3 Faculty of Dentistry, Chulalongkorn University, Bangkok, Thailand

**Keywords:** dentist, dentistry, remote/rural, self-perception, teledentistry, telemedicine

## Abstract

**Objectives:**

This study aims to explore the beliefs and attitudes related to the adoption of teledentistry among Pakistani dental professionals, focusing on data security, practice enhancement, and patient benefits.

**Material and Methods:**

A cross-sectional study on a 5-point Likert scale assessed four domains of teledentistry: data security and patient consent, practice improvement capabilities, usefulness for dental practice, and patient benefits, among dental professionals through electronic forms. Demographic data and items from four domains were analyzed by descriptive statistics, analysis of variance and Pearson's correlation tests, respectively, using SPSS, with a *p* -value of < 0.05 set as statistically significant.

**Results:**

A large percentage (59.8%) of the 408 dental professionals raised issues related to data security with 52% showing concerns about securing patient consent. Most professionals (61.8%) acknowledged the potential of teledentistry in reducing waiting times. Gender, age, qualifications, and work experience were found to be associated with individual perceptions of teledentistry. Females were more skeptical on teledentistry capabilities ( *p* < 0.000) while younger, more than the older professionals had heightened worries about data security ( *p* < 0.000). Specialists viewed teledentistry favorably compared with other professionals ( *p* < 0.000). Professionals with more than 5 years of experience expressed optimism, on how teledentistry could improve practice efficiency and patient outcomes ( *p* < 0.000).

**Conclusion:**

This report on the views and attitudes of Pakistani dental professionals toward teledentistry indicates their positive perception of teledentistry, citing its potential to enhance practice and benefit patients. Overcoming data security concerns and improving education on teledentistry benefits could foster broader acceptance and utilization of this technology.

## Introduction

Teledentistry can be defined as the utilization of advanced technology to provide remote dental care and consultation. In recent years, there has been a significant evolution in the approach to dental care with the emergence of teledentistry. 1 Teledentistry essentially entails utilization of advanced digital technologies for offering remote dental care, consultation, and services. 2 Thus, teledentistry will facilitate dental care in people living in remote rural areas with limited access to dental services.

For instance, instead of traveling long distances for checkups that are costly, patients are now able to consult their dentists using teledentistry tools such as online consultations or video calls from the comfort of their homes or local clinics where such facilities are available. 3 Teledentistry also plays a crucial role in facilitating teleconsultations and reducing waiting times for dental care by enabling off-site decision-making and treatment planning, with remote, streamlined appointments. This leads to shorter appointment durations, ultimately expediting oral health assistance for patients in need. 4 5 6 7 Furthermore, teledentistry could be used for dental education eventually resulting in improved patient care. 8 However, many practitioners are unaware of its benefits for their clinical practice and patient health. 9 These benefits include increased access to care, enhanced productivity, and efficient treatment delivery to underserved populations. 8

In developing jurisdictions, mainly in Asia, the utilization of teledentistry, despite its advantages, has been limited. Challenges reported include obtaining approvals from authorities, financing infrastructure, and technology setup. 10 Technical issues including slow Internet and connectivity problems pose significant hurdles, 11 and patient privacy concerns and varying levels of technology literacy further complicate matters. 12 There are also some risks associated with remote consultations, such as potential failure to capture all patient complaints or misinterpretation of communications. 13

Several studies including those from Australia, 14 Canada, 15 Saudi Arabia, 16 and France 17 have been conducted at a national level to explore perceptions, effectiveness, and utilization of teledentistry. Nonetheless, there is a critical gap in applying these findings to other geographic regions, particularly South Asia. The lack of sufficient data from this region limits our understanding of teledentistry applicability and effectiveness. Therefore, bridging this gap becomes essential.

In Pakistan, a country the size of over 881,913 square kilometers (the size of France and the United Kingdom combined) with numerous remote and inaccessible areas, the delivery of dental care faces challenges due to its vast and remote terrain. The population's sparse distribution contributes to issues such as a shortage of dentists, unaffordability of dental care, and long distances to health care facilities providing dental services. 18 The Pakistan government's National Health Vision 2025, 19 aimed at enhancing the nation's health by improving access to quality health care services and utilizing information technology in the sector, now presents opportunities for the development of teledentistry in the country. To effectively implement teledentistry and align with such a national vision, it is crucial to understand the knowledge, awareness, and attitudes of dental professionals in Pakistan toward teledentistry, as well as the perceived challenges in its implementation. However, there has been a lack of studies evaluating the level of awareness of teledentistry among dental personnel in Pakistan. Obtaining this information will provide valuable insights for planning and implementing teledentistry initiatives in the region.

Hence, the purpose of this study was to analyze the knowledge, awareness, and attitudes related to teledentistry, in a cohort of dental professionals working in the city of Karachi, Pakistan.

## Materials and Methods

This study was conducted from November 2023 through April 2024 among undergraduate and postgraduate dental professionals, postgraduate trainees, and dental hygienists. The sample size was calculated 20 as follows: *n* = *z* ^2^ × *p* × [1 − *p* ]/ *e* ^2^ ( *z* = 1.96 for a confidence level of 95%, *p* = proportion [50% expressed as a decimal 0.50], *e* = margin of error [0.05]), thus, *n* = 1.96 ^2^ × 0.5 × (1–0.5)/0.05 2 = 384.16 ≈ 385. A convenient sampling method was used for the study. Approval was obtained from the Institutional Review Board of Muhammad Dental College (Reference number: MDC/0713) to conduct the study.

**Fig. 1:**
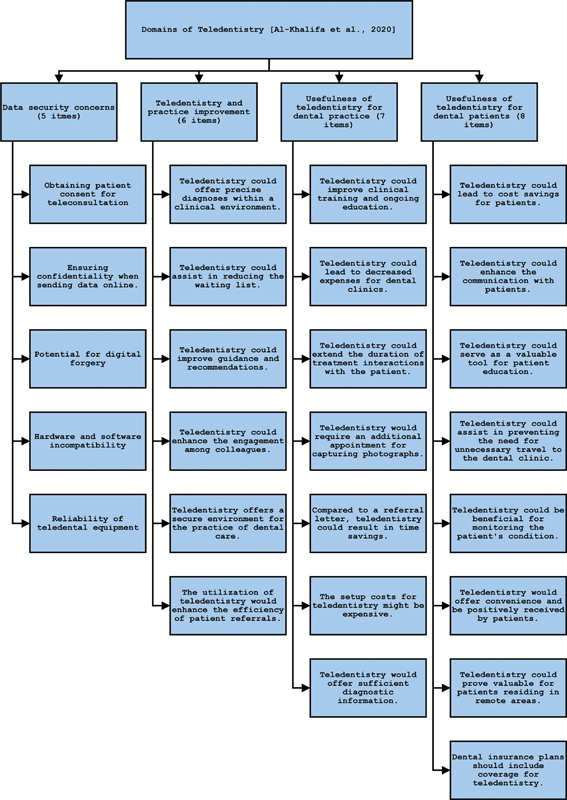
The evaluated domains of teledentistry and their subsets.

The study involved distributing questionnaires via Google Form to dental professionals in Karachi city of Sindh province, Pakistan within our network using WhatsApp and email. These participants were requested to share the Google Form link with other dental professionals to broaden participation. The questionnaire was an adaptation modified from a previous study by Al-Khalifa and AlSheikh, and included demographic and professional data, preferred communication methods in teledentistry, and four separate domains of teledentistry, including (1) data security and patient consent, (2) the potential of teledentistry to improve dental care, (3) usefulness of teledentistry for dental practice, and (4) usefulness of teledentistry for patients ( Fig. 1 ).

In response to reviewer feedback, we classified the questionnaire items based on “knowledge,” “awareness,” and “attitude,” as shown in Supplementary Table S1 (available in the online version only). This classification helped clarify the focus of each item with our original domains.

Each domain comprised multiple items rated on a 5-point Likert scale ranging from a score of 1 for not concerned at all, to 5 for extremely concerned. The questionnaire began with an introduction to teledentistry, its advantages, applications, and a consent agreement outlining the study's purpose. All responses were collected anonymously to ensure confidentiality, and permission from institutions or clinics was not sought, as participants were approached as individuals.

## Data Analysis

**Fig. 2:**
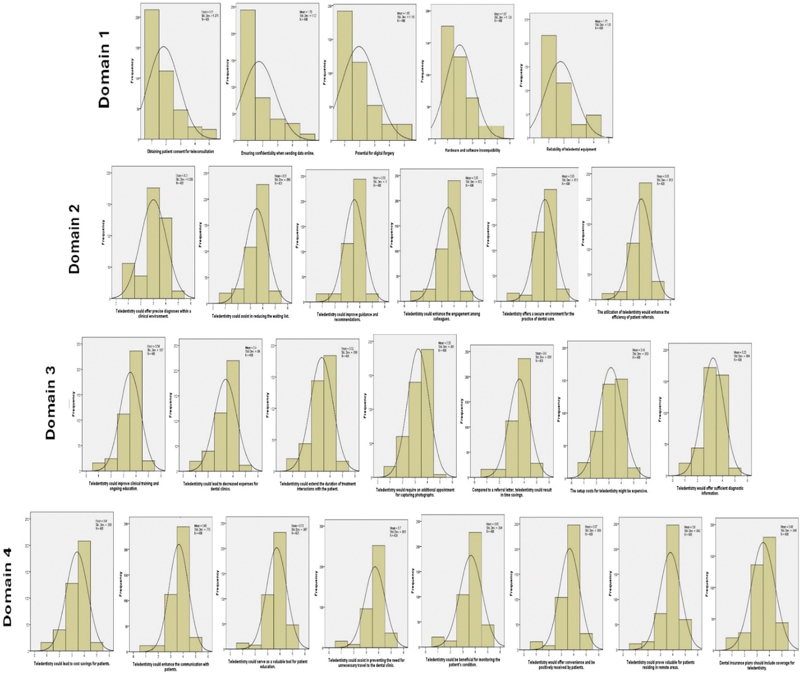
Frequency of the items evaluated in the four domains of teledentistry.

Data was downloaded from Google Forms into MS Excel 2016 for coding based on a 5-point Likert scale, then analyzed using IBM SPSS version 29. A pilot test with 20 dentists was conducted to determine internal consistency using Cronbach's *α* , yielding a satisfactory value of 0.85. These were not included in the final analysis of the study. Descriptive statistics analyzed means, standard deviations, and frequencies for demographic variables and the four domains of teledentistry. A normality test confirmed the data were normally distributed ( Fig. 2 ). Analysis of variance (ANOVA) was used to analyze mean score differences of independent variables, and Pearson's correlation assessed the linear correlation between all domains, with *p* -values less than 0.05 considered significant.

## Results

### Demographic Data

The final cohort sampled were 408 dental professionals and comprised 80.4% ( *n* = 328) females. A vast majority (83.3%; *n* = 340) fell within the 20 to 34 age group. General dentists constituted the largest cohort (66.7%; *n* = 272), followed by residents (15.7%; *n* = 64). The majority of participants (69.6%; *n* = 284) reported having 0 to 5 years of experience. A significant proportion (58.8%, *n* = 240) were engaged in private practice. Note that 44.1% ( *n* = 180) reported working 1 to 19 hours per week ( Table 1 ).

**Table 1:** Demographic data

Variables		Frequency ( *n* )	%
Gender	Male	80	19.6
	Female	328	80.4
			
Age group	20–34	340	83.3
	35–44	60	14.7
	45–54	8	2.0
	> 55	0	0
			
Qualification	General dental practitioner	272	66.7
	Resident/Graduate	64	15.7
	Specialist	34	8.3
	Dental hygienist/assistant	38	9.3
			
			
Work experience (in years)	0–5	284	69.6
	6–10	72	17.6
	11–15	40	9.8
	> 16	12	2.9
			
Work setting	Private	240	58.8
	Governmental	64	15.7
	Both (private and governmental)	32	7.8
	Academic	44	10.8
	No job	28	6.9
			
Working hours per week	1–19 h	180	44.1
	20–34 h	76	18.6
	35–49 h	84	20.6
	50–64 h	54	13.2
	> 65 h	14	3.4
			
Daily use of the Internet for general purposes (in hours)	< 1	24	5.9
	2–4	240	58.8
	5–7	124	30.4
	8–10	20	4.9
	> 11	0	0.0
			
Daily use of the Internet in dental practice (in hours)	< 1	224	54.9
	2–4	152	37.3
	5–7	28	6.9
	8–10	4	1.0
	> 11	0	0.0

**Fig. 3:**
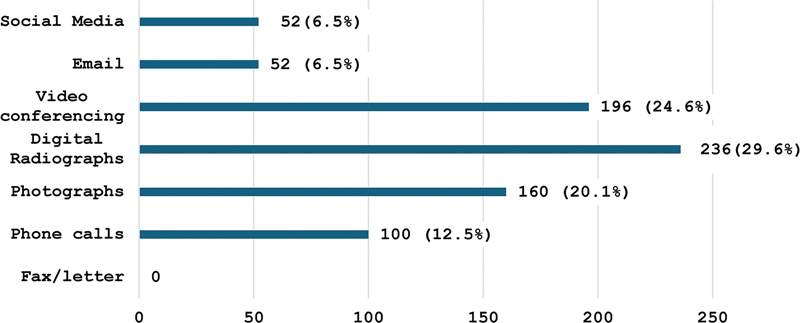
Preferred communication tool for teledentistry among dental professionals.

The preferred communication tools for teledentistry among dental professionals revealed interesting trends ( Fig. 3 ). Thus, only a minority, 29.6% ( *n* = 236), preferred digital radiographs, followed by photographic images (20.1%; *n* = 160) and teleconference participation (24.6%; *n* = 196), and finally, phone calls (12.5%; *n* = 100). Email and social media were the least preferred media tools, each chosen by 6.5% ( *n* = 52). Fax/letters were not selected by any dental professionals.

### Domain 1: Data Security Concerns

The concerns of dental professionals on teledentistry including confidentiality and security are shown in Table 2 . The most significant concern expressed by the majority (59.8%; *n* = 244) was ensuring patient data confidentiality online, while a fifth of the respondents (19.6%; *n* = 80) were somewhat concerned. Obtaining patient consent for teleconsultation was another major concern, with 52.0% ( *n* = 212) very concerned and 27.5% ( *n* = 112) somewhat concerned. Concerns about hardware and software incompatibility were reported by 43.1% ( *n* = 176), while 31.4% ( *n* = 128) were somewhat concerned. Finally, the reliability of teledental equipment was a significant issue, with 52.9% ( *n* = 216) very concerned and 28.4% ( *n* = 116) somewhat concerned.

**Table 2:** Responses of domain 1 to 4 items among dental professionals ( *n* = 408)

Domain 1. Understanding of data security and patient consent						
Items		Very concerned	Little concerned	Not feeling either way	Not particularly concerned	Not concerned at all
Obtaining patient consent for teleconsultation	Count Row, *N* %	212 52	112 27.5	48 11.8	20 4.9	16 3.9
Ensuring confidentiality when sending data online	Count Row, *N* %	244 59.8	80 19.6	40 9.8	32 7.8	12 2.9
Potential for digital forgery	Count Row, *N* %	192 47.1	116 28.4	52 12.7	24 5.9	24 5.9
Hardware and software incompatibility	Count Row, *N* %	176 43.1	128 31.4	64 15.7	20 4.9	20 4.9
Reliability of teledental equipment	Count Row, *N* %	216 52.9	116 28.4	28 6.9	48 11.8	0 0
**Domain 2. Capability of Teledentistry to Improve Practice**						
**Items**		**Disagree strongly**	**Disagree**	**Neutral**	**Agree**	**Agree strongly**
Teledentistry could offer precise diagnoses within a clinical environment	Count Row, *N* %	56 13.7	36 8.8	176 43.1	128 31.4	12 2.9
Teledentistry could assist in reducing the waiting list	Count Row, *N* %	20 4.9	28 6.9	108 26.5	228 55.9	24 5.9
Teledentistry could improve guidance and recommendations	Count Row. *N* %	16 3.9	16 3.9	116 28.4	244 59.8	16 3.9
Teledentistry could enhance engagement among colleagues	Count Row, *N* %	20 4.9	24 5.9	104 25.5	240 58.8	20 4.9
Teledentistry offers a secure environment for the practice of dental care	Count Row, *N* %	16 3.9	12 2.9	136 33.3	220 53.9	24 5.9
The utilization of teledentistry would enhance the efficiency of patient referrals	Count Row, *N* %	12 2.9	16 3.9	112 27.5	232 56.9	36 8.8
**Domain 3. Teledentistry Usefulness for Dental Practice**						
**Items**		**Disagree strongly**	**Disagree**	**Neutral**	**Agree**	**Agree strongly**
Teledentistry could improve clinical training and ongoing education	Count Row, *N* %	16 3.9	24 5.9	112 27.5	236 57.8	20 4.9
Teledentistry could lead to decreased expenses for dental clinics	Count Row, *N* %	20 4.9	40 9.8	116 28.4	220 53.9	12 2.9
Teledentistry could extend the duration of treatment interactions with the patient	Count Row, *N* %	20 4.9	44 10.8	144 35.3	184 45.1	16 3.9
Teledentistry would require an additional appointment for capturing photographs	Count Row, *N* %	16 3.9	60 14.7	140 34.3	188 46.1	4 1.0
Compared with a referral letter, teledentistry could result in time savings	Count Row, *N* %	16 3.9	16 3.9	112 27.5	236 57.8	28 6.9
The setup costs for teledentistry might be expensive	Count Row, *N* %	24 5.9	72 17.6	144 35.3	152 37.3	16 3.9
Teledentistry would offer sufficient diagnostic information	Count Row, *N* %	20 4.9	44 10.8	172 42.2	160 39.2	12 2.9
**Domain 4. Teledentistry Usefulness for Dental Patients**						
**Items**		**Disagree strongly**	**Disagree**	**Neutral**	**Agree**	**Agree strongly**
Teledentistry could lead to cost savings for patients	Count Row, *N* %	16 3.9	40 9.8	128 31.4	208 51	16 3.9
Teledentistry could enhance the communication with patients	Count Row, *N* %	12 2.9	12 2.9	112 27.5	244 59.8	28 6.9
Teledentistry could serve as a valuable tool for patient education	Count Row, *N* %	12 2.9	8 2	108 26.5	232 56.9	48 11.8
Teledentistry could assist in preventing the need for unnecessary travel to the dental clinic	Count Row, *N* %	16 3.9	8 2	96 23.5	252 61.8	36 8.8
Teledentistry could be beneficial for monitoring the patient's condition	Count Row, *N* %	20 4.9	12 2.9	104 25.5	228 55.9	44 10.8
Teledentistry would offer convenience and be positively received by patients	Count Row, *N* %	16 3.9	8 2	104 25.5	248 60.8	32 7.8
Teledentistry could prove valuable for patients residing in remote areas	Count Row, *N* %	12 2.9	16 3.9	72 17.6	248 60.8	60 14.7
Dental insurance plans should include coverage for teledentistry	Count Row, *N* %	20 4.9	28 6.9	136 33.3	180 44.1	44 10.8

Note: Descriptive statistics.

### Domain 2: Capability of Teledentistry to Improve Practice

The potential for teledentistry to offer precise diagnoses elicited varied responses, with 43.1% ( *n* = 176) being neutral, 31.4% ( *n* = 128) agreeing, and 2.9% ( *n* = 12) strongly agreeing on this element. Regarding the reduction of waiting lists, a majority viewed teledentistry positively, with 55.9% ( *n* = 228) agreeing and 5.9% ( *n* = 24) strongly agreeing. The improvement of guidance and recommendations through teledentistry was supported by 59.8% ( *n* = 244) of participants agreeing.

Enhancing engagement among colleagues was another perceived benefit, with 58.8% ( *n* = 240) agreeing. In terms of offering a secure environment for dental care, 53.9% ( *n* = 220) affirmed this to be the case. Finally, the use of teledentistry to enhance the efficiency of patient referrals was positively received, with 56.9% ( *n* = 232) agreeing with this issue ( Table 2 ). These results indicated that dental professionals generally perceived teledentistry as having the potential to enhance differing aspects of dental practice.

### Domain 3: Teledentistry Usefulness for Dental Practice

The opinions of dental professionals on the effectiveness of teledentistry in dental practice are presented in Table 2 . Accordingly, 57.8% ( *n* = 236) viewed teledentistry as a very positive medium for advancing clinical training and continuing education. Additionally, 53.9% (220) agreed that teledentistry could decrease dental clinic expenditure, while 45.1% ( *n* = 184) believed that teledentistry will be of use for extending interaction duration with patients.

A significant percentage of participants (57.8%; *n* = 236) perceived that teledentistry saves time compared with referral letters. Concerns regarding the expensive setup costs for teledentistry were acknowledged by 37.3% ( *n* = 152) of respondents. On the other hand, 39.2% ( *n* = 160) had a positive view of the diagnostic information provided by teledentistry, considering it sufficient.

### Domain 4: Teledentistry Usefulness for Dental Patients

The perspectives of dental professionals on the implementation and advantages of teledentistry for dental professionals are presented in Table 2 . Just over one-half of the respondents (51%; *n* = 208) thought that teledentistry can lead to cost savings for patients, and improve communication with patients (59.8%; *n* = 244). Additionally, 56.9% considered it to be a valuable tool for patient education, and 61.8% ( *n* = 252) acknowledged its role in reducing unnecessary travel to the dental clinic.

Furthermore, 55.9% ( *n* = 228) acknowledged its benefits for monitoring patients health, and 60.8% agreed the convenience of the technology and positive reception by patients. A majority, 60.8% ( *n* = 248), also recognized its value for patients residing in remote areas, while 44.1% ( *n* = 180) believed that dental insurance plans should include coverage for teledentistry.

### Comparisons in the Mean Scores of Independent Variables across the Four Domains of Teledentistry

ANOVA test was used to analyze the demographic variables versus the dental professionals' perceptions of teledentistry ( Table 3 ). Significant differences were found based on gender and age in the domain of data security and patient consent ( *p* < 0.000), with females and older participants expressing higher concerns. In terms of the capability of teledentistry to improve practice, specialists rated it higher than residents and general dentists ( *p* < 0.000). Those with more work experience rated teledentistry lower across most variables.

**Table 3:** Dental professionals' demographic variables related to the evaluated four domains of teledentistry

Variables	Data security and patient consent, mean (SD)	The capability of teledentistry to improve practice, mean (SD)	The usefulness of teledentistry for dental practice, mean (SD)	The usefulness of teledentistry for patients, mean (SD)
Gender				
Male	1.55 ± 0.62	3.41 ± 0.92	3.10 ± 0.86	3.50 ± 0.92
Female	1.92 ± 0.90	3.47 ± 0.57	3.42 ± 0.54	3.66 ± 0.60
*p* -Value	< 0.001 a	0.441	< 0.001 a	0.047
Age group				
20–34	1.94 ± 0.89	3.50 ± 0.58	3.39 ± 0.55	3.66 ± 0.59
35–44	1.42 ± 0.52	3.24 ± 0.95	3.13 ± 0.94	3.50 ± 1.07
45–54	1.20 ± 0.21	3.58 ± 0.89	3.42 ± 0.15	3.56 ± 0.20
*p* -Value	< 0.001 a	0.016 a	0.010 a	0.228
Qualification				
Resident/Graduate	2.03 ± 0.91	3.51 + 0.49	3.39 ± 0.41	3.73 ± 0.47
General dentist	1.87 ± 0.88	3.39 ± 0.69	3.33 ± 0.65	3.54 ± 0.74
Specialist	1.68 ± 0.77	3.88 ± 0.49	3.45 ± 0.75	4.08 ± 0.43
Dental hygienist	1.50 ± 0.53	3.54 ± 0.54	3.37 ± 0.60	3.68 ± 0.49
*p* -Value	0.013 a	< 0.001 a	0.737	< 0.001 a
Work experience (years)				
0–5	1.94 ± 0.90	3.49 ± 0.54	3.41 ± 0.50	3.65 ± 0.60
6–10	1.87 ± 0.76	3.38 ± 0.87	3.19 ± 0.86	3.56 ± 0.82
11–15	1.34 ± 0.48	3.63 ± 0.43	3.51 ± 0.49	3.86 ± 0.45
> 16	1.13 ± 0.19	2.83 ± 1.37	2.76 ± 1.30	2.91 ± 1.41
*p* -Value	< 0.001 a	< 0.001 a	< 0.001 a	< 0.001 a

Abbreviation: ANOVA, analysis of variance; SD, standard deviation.

a ANOVA, *α* = 0.05,

Pearson's correlation analysis indicated a strong positive correlation between the domains related to the capability of teledentistry to improve practice and its usefulness for dental practice and patients ( *p* < 0.000; Table 4 ).

**Table 4:** Pearson's correlation among each of the domains evaluated clinical parameters

Domains		Domain 1	Domain 2	Domain 3	Domain 4
Domain 1	Pearson's correlation	−	0.068	0.001	-0.089
Domain 2	Pearson's correlation	0.068	−	0.742 a	0.796 a
Domain 3	Pearson's correlation	0.001	0.742 a	−	0.754 a
Domain 4	Pearson's correlation	–0.089	0.796 a	0.754 a	−

Domain 1: Data security and patient consent.

Domain 2: The capability of teledentistry to improve practice.

Domain 3: The usefulness of teledentistry for dental practice.

Domain 4: The usefulness of teledentistry for patients.

a Correlation is significant at the *p* -value < 0.05.

**Fig. 4:**
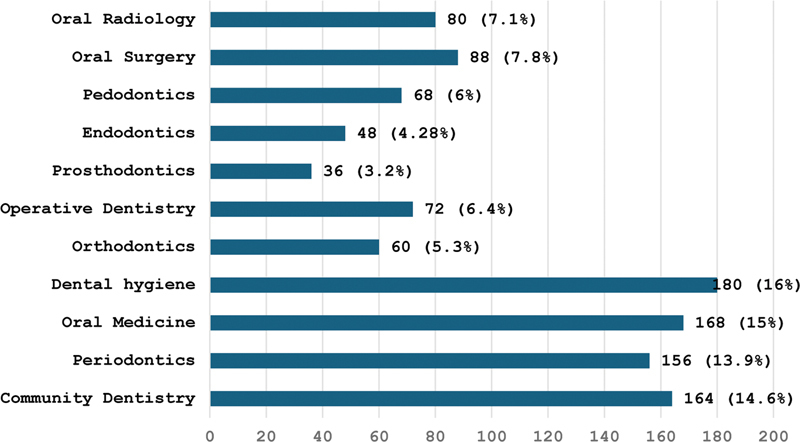
Responses on the application of teledentistry in different dental disciplines.

With regard to clinical application of teledentistry varied views were noted among specialties, with dental hygienists showing the highest interest, in contrast to low interest shown by oral surgeons and prosthodontists ( Fig. 4 ). On the other hand, periodontics, oral medicine, orthodontics, operative dentistry, and endodontics personnel showed a moderate interest in utilizing teledentistry. Finally, our data should help policymakers and health care providers in Pakistan develop tailored strategies to address concerns, such as data security, and maximize the benefits of teledentistry across diverse user groups.

## Discussion

This survey represents one of the first attempts to investigate the perception of teledentistry among dental professionals in Pakistan. Access to such technology, especially when diseases such as coronavirus disease 2019 and various viral infections are spreading in the community, has led to the realization by the dental professionals of the importance of teledentistry as a highly useful clinical communication tool. This is particularly relevant in countries like Pakistan, where a significant proportion of the population resides in remote areas, far from major urban centers, and lacks adequate access to dental care.

The study findings unveiled significant variations in perceptions toward different aspects of teledentistry. Gender was identified as a notable determinant, with females expressing heightened concerns regarding data security and patient consent. This observation aligns with prior research that highlights gender differences in technology adoption and privacy concerns among health care professionals in general. 21 22

Age was also found to play a role, as older professionals exhibited lower levels of concern but higher perceptions of teledentistry's potential to enhance dental practice and benefit patients. These age-related differences reflect findings from previous studies emphasizing generational variations in technology acceptance and perceived benefits within health care contexts. 23 24

The analysis of teledentistry domains revealed nuanced perspectives among dental professionals. Concerns related to confidentiality and security issues were prevalent, with participants expressing varying levels of apprehension, particularly on obtaining patient consent and ensuring confidentiality when sending data online. Similar findings have been reported in studies investigating health care professionals' perceptions of telemedicine and data security where 80 to 84% of the respondents worried about the confidentiality of patient data when transmitted online. 25 26 Issues such as digital forgery (90%), incompatible software and hardware, and the accuracy of equipment (80%) were also major concerns in another study. 27 However, there was a strong consensus on the potential benefits of teledentistry for improving practice efficiency, reducing waiting lists, enhancing guidance and recommendations, and offering a secure environment for dental care in all the studies including ours. These positive perceptions align with literature highlighting the transformative impact of telehealth technologies on health care delivery and patient outcomes. 28 29 30 31

Integrating multiple communication platforms can cater to varied preferences, enhance the adoption of teledentistry, and improve remote dental care. Our data showed diverse preferences for communication tools in teledentistry, with dental professionals favoring digital radiographs, video conferencing, and photos, highlighting a clear shift toward more modern, digital communication methods in teledentistry. In a previous study, 16 and 22% of respondents preferred audio calls and email, respectively. 32

The ANOVA analyses conducted on independent variables (gender, age, qualification, and duration of work experience) revealed statistically significant associations with perceptions of teledentistry across different domains. Specialists demonstrated higher levels of perceived capability and usefulness of teledentistry compared with general dentists and residents, suggesting a greater enthusiasm for integrating teledentistry into clinical practice. In another study, 32 residents/graduate researchers rated data security and patient consent the highest, and dental therapists rated the capability to improve practice the highest. Female participants perceived teledentistry as more useful for patients compared with males, highlighting potential gender-related differences in the perceived benefits of teledentistry. These findings align with studies exploring health care professionals' attitudes and adoption of telehealth technologies based on professional roles and gender differences. 33 34

Moreover, Pearson's correlation analysis between all four domains revealed a strong and positive correlation between domains 2 and 4 (capability of teledentistry to improve practice, teledentistry usefulness for dental practice, and teledentistry usefulness for dental patients), which aligns with a previous study by Al-Khalifa and AlSheikh. 32

Our findings have several implications for the successful implementation of teledentistry in dental practice particularly in Pakistan. Addressing specific concerns related to data security, confidentiality, and patient consent is crucial for fostering trust and acceptance among dental professionals. Emphasizing the practice-enhancing effects and patient benefits of teledentistry can promote its adoption and utilization across different professional groups. Additionally, integrating preferred communication tools identified by participants can optimize the usability and effectiveness of teledentistry platforms in clinical settings. These implications aligned with the recommendations reported in studies emphasizing the importance of addressing barriers and adapting interventions to promote the adoption of telehealth technologies in health care. 35 36 37 38

## Study Limitations and Future Directions

Our study has some limitations. First, the sample size was small, not randomized, and limited to Karachi and second, the regional-specific study may limit its generalizability to the whole country. A bigger sample size from various regions of Pakistan would have resulted in more country-specific data. Additionally, relying on information provided by the participants themselves, the research might be biased if they give socially preferred answers rather than expressing what they believe and what they do in reality. Further studies with qualitative methods are needed to determine various other factors influencing the use of teledentistry, such as technological literacy, access to technology, and specific barriers, compare the perception of teledentistry with traditional dental practice, and to look at how the views and experiences of dental professionals change with time as teledentistry progresses and becomes more integrated into everyday practice, and finally, longitudinal studies will also be of interest.

## Conclusion

Teledentistry was viewed favorably by dental professionals, mainly female and young, although there were concerns about patient consent, data privacy, and implementation costs. Teledentistry has been perceived as useful for accurate diagnosis, reduction of waiting times, improvement of guidance, as well as the advancement of clinical education and communication. We noted teledentistry was preferred by less experienced, female and younger professionals. To ensure a seamless transition to the use of teledentistry, future research should attempt to address the issues and obstacles found in this study.
