# Supplementary material for: Perception and Attitudes of Dental Professionals on Teledentistry: A Cross-Sectional Study
Source: Eur J Dent. 2025 Mar 12;19(4):1134–45. doi: 10.1055/s-0044-1801301 (PMC12494421; doi:10.1055/s-0044-1801301)
Supplement: Supplementary file 1 — Supplementary Material [file 10-1055-s-0044-1801301-s24103826.pdf]

Supplementary Table S1 Classification of teledentistry questionnaire domain items by knowledge, awareness, and attitude

Original questionnaire items	Domain	Classification
Obtaining patient consent for teleconsultation	Data security and patient consent	Knowledge
Ensuring confidentiality when sending data online		
Potential for digital forgery		
Hardware and software incompatibility		
Reliability of teledental equipment		
Teledentistry could offer precise diagnoses within a clinical environment	Capability of Teledentistry to Improve dental Practice	Awareness
Teledentistry could assist in reducing the waiting list		
Teledentistry could improve guidance and recommendations		
Teledentistry could enhance the engagement among colleagues		
Teledentistry offers a secure environment for the practice of dental care		
The utilization of teledentistry would enhance the efficiency of patient referrals		
Teledentistry could improve clinical training and ongoing education	Teledentistry Usefulness for Dental Practice	Attitude
Teledentistry could lead to decreased expenses for dental clinics		
Teledentistry could extend the duration of treatment interactions with the patient		
Teledentistry would require an additional appointment for capturing photographs		
Compared with a referral letter, teledentistry could result in time savings		
The setup costs for teledentistry might be expensive		
Teledentistry would offer sufficient diagnostic information		
Teledentistry could lead to cost savings for patients	Usefulness of teledentistry for patients	Awareness
Teledentistry could lead to cost savings for patients		
Teledentistry could serve as a valuable tool for patient education		
Teledentistry could assist in preventing the need for unnecessary travel to the dental clinic		
Teledentistry could be beneficial for monitoring the patient's condition		
Teledentistry would offer convenience and be positively received by patients		
Teledentistry could prove valuable for patients residing in remote areas		
Dental insurance plans should include coverage for teledentistry		

Note: Knowledge refers to factual information about teledentistry.
Awareness reflects understanding of teledentistry's potential applications.
Attitude represents perceptions and beliefs regarding the usefulness and feasibility of teledentistry.

 © 2025. The Author(s).
